# Comprehensive Brain Tumour Characterisation with VERDICT-MRI: Evaluation of Cellular and Vascular Measures Validated by Histology

**DOI:** 10.3390/cancers15092490

**Published:** 2023-04-27

**Authors:** Matteo Figini, Antonella Castellano, Michele Bailo, Marcella Callea, Marcello Cadioli, Samira Bouyagoub, Marco Palombo, Valentina Pieri, Pietro Mortini, Andrea Falini, Daniel C. Alexander, Mara Cercignani, Eleftheria Panagiotaki

**Affiliations:** 1 Centre for Medical Image Computing and Department of Computer Science, University College London, London WC1V 6LJ, UK; 2 Neuroradiology Unit and CERMAC, IRCCS Ospedale San Raffaele, Vita-Salute San Raffaele University, 20132 Milan, Italy; 3 Department of Neurosurgery and Gamma Knife Radiosurgery, IRCCS Ospedale San Raffaele, Vita-Salute San Raffaele University, 20132 Milan, Italy; 4 Pathology Unit, IRCCS Ospedale San Raffaele, 20132 Milan, Italy; 5 Philips Healthcare, 20126 Milan, Italy; 6 Clinical Imaging Sciences Centre, Brighton and Sussex Medical School, Brighton BN1 9RR, UK; 7 Cardiff University Brain Research Imaging Centre (CUBRIC), School of Psychology, Cardiff University, Cardiff CF24 4HQ, UK

**Keywords:** MRI, diffusion MRI, VERDICT, brain tumours, microstructure, biophysical model, multi-compartment, cell density, vascularity, oedema

## Abstract

**Simple Summary:**

VERDICT (Vascular, Extracellular, and Restricted DIffusion for Cytometry in Tumours) is a diffusion MRI framework for the characterisation of different components of tumours, which has shown diagnostic utility for body cancer. The aim of this study was to extend the VERDICT framework to comprehensively characterise brain tumours, which is challenging due to the complexity of brain tissues. The resulting biomarkers showed agreement with histology and followed the expected trends when comparing different tumour types and sub-regions. These preliminary results hold promise for the non-invasive characterisation of brain tumours by VERDICT-MRI, which would be an important tool for diagnosis and monitoring of treatment effects.

**Abstract:**

The aim of this work was to extend the VERDICT-MRI framework for modelling brain tumours, enabling comprehensive characterisation of both intra- and peritumoural areas with a particular focus on cellular and vascular features. Diffusion MRI data were acquired with multiple b-values (ranging from 50 to 3500 s/mm^2^), diffusion times, and echo times in 21 patients with brain tumours of different types and with a wide range of cellular and vascular features. We fitted a selection of diffusion models that resulted from the combination of different types of intracellular, extracellular, and vascular compartments to the signal. We compared the models using criteria for parsimony while aiming at good characterisation of all of the key histological brain tumour components. Finally, we evaluated the parameters of the best-performing model in the differentiation of tumour histotypes, using ADC (Apparent Diffusion Coefficient) as a clinical standard reference, and compared them to histopathology and relevant perfusion MRI metrics. The best-performing model for VERDICT in brain tumours was a three-compartment model accounting for anisotropically hindered and isotropically restricted diffusion and isotropic pseudo-diffusion. VERDICT metrics were compatible with the histological appearance of low-grade gliomas and metastases and reflected differences found by histopathology between multiple biopsy samples within tumours. The comparison between histotypes showed that both the intracellular and vascular fractions tended to be higher in tumours with high cellularity (glioblastoma and metastasis), and quantitative analysis showed a trend toward higher values of the intracellular fraction (fic) within the tumour core with increasing glioma grade. We also observed a trend towards a higher free water fraction in vasogenic oedemas around metastases compared to infiltrative oedemas around glioblastomas and WHO 3 gliomas as well as the periphery of low-grade gliomas. In conclusion, we developed and evaluated a multi-compartment diffusion MRI model for brain tumours based on the VERDICT framework, which showed agreement between non-invasive microstructural estimates and histology and encouraging trends for the differentiation of tumour types and sub-regions.

## 1. Introduction

Characterising the microstructure of brain tumours and peritumoural space is fundamental to providing accurate preoperative diagnosis and selecting the most suitable treatment option for each patient, aiming at tailoring individualised therapies. Achieving this non-invasively, however, is still a challenge in current clinical practice. Specifically, what remains elusive is the reliability of non-invasive imaging methods to consistently unravel biological and microstructural features of the tumour tissue [1]. As not all patients are eligible for biopsy or surgical intervention, which is currently required for definitive histopathological and molecular diagnosis, it is essential to implement methodologies to validate harmless diagnostic and predictive imaging biomarkers for brain tumours [2]. A non-invasive definition of microscopic tissue modifications over time could enable assessment of surrogate indicators of disease response or progression, avoiding the intrusive need of repeated biopsies during patient follow-up [3].

The MRI protocol for the diagnosis and management of brain tumours recommended by the European Organisation for Research and Treatment of Cancer and the National Brain Tumour Society (EORTC-NBTS consensus recommendations) includes mainly morphological sequences [4]: lesions are commonly detected on T2-weighted or Fluid-Attenuated Inversion Recovery (FLAIR) images, and T1-weighted images acquired after the injection of gadolinium-based contrast agents are used to identify blood–brain barrier damage. Even though recent radiomics approaches have shown great value for differentiating tumour types or monitoring treatment effects even just with morphological MRI [5,6], this basic characterisation is often not sufficient [7]. Advanced MRI techniques, such as Diffusion MRI (dMRI) and Perfusion-Weighted Imaging (PWI), are increasingly included in both research and clinical MRI protocols for brain tumours, as they add important structural, physiological, and haemodynamic information [8]. Other imaging modalities, such as Positron Emission Tomography (PET), have shown very good diagnostic performance and sensitivity for the metabolic characterisation of brain tumours both at initial diagnosis and during follow-up [9,10,11]. However, the use of radioactive tracers makes PET less suitable than MRI in some patients, especially when multiple follow-up scans are needed.

Among advanced MRI techniques, dMRI is sensitive to the cellular microenvironment of healthy brain tissues and brain tumours [12]. In its basic form, dMRI provides the Apparent Diffusion Coefficient (ADC), which has been shown to inversely correlate with cellularity [13,14,15]. Thanks to its sensitivity, dMRI for ADC computation is included in the recommended brain tumour imaging protocol [4], even though some contradictory findings were reported [16] and tumour-specific ranges of ADC values considerably overlap [17]. Diffusion Kurtosis Imaging (DKI), a higher-order analysis of dMRI data, has been shown to be more sensitive and accurate in grading and differentiating brain tumours [18,19], based on the Mean Kurtosis (MK) parameter, which is related to diffusional variance. Recent studies used tensor-valued diffusion encoding MRI acquisitions to decompose MK into two metrics associated with microscopic anisotropy and tissue heterogeneity, respectively, which were applied to differentiate brain tumour types [20,21]. Another approach is to fit a multi-compartment model [22] separating the signal contributions coming from different tissue types (or components); a certain level of complexity is needed for these models to be biologically relevant, which implies a high risk of degeneracy [23]. A common solution is to fix or constrain some of the model parameters, such as in Neurite Orientation Dispersion and Density Imaging (NODDI) [24], which has been applied in brain tumours [25,26,27] even though its assumptions are not appropriate for the whole tumour area. A better alternative is to acquire richer imaging datasets, for example with multiple diffusion times or oscillating gradients, which provide model stability while allowing for measurements of cell size and density [28].

The dMRI methods mentioned so far focus on the cellular component of brain tumours and neglect vasculature. dMRI can also characterise microvascularity by modelling perfusion in vessels as pseudo-diffusion (with very high diffusivity). Contrary to common PWI techniques, such as Dynamic Contrast-Enhanced (DCE) or Dynamic Susceptibility Contrast (DSC) MRI [29,30], dMRI does not require contrast agent injection. The first diffusion model to account for perfusion effects was the Intra-Voxel Incoherent Motion (IVIM) model [31]. IVIM has been applied to brain tumours (see for example [32,33]), showing good prognostic value for response to therapy or progression and good correlation with DSC and DCE metrics. However, standard IVIM characterisation of the cellular components of tissues is based on simple monoexponential decay and does not account for restriction, anisotropy, or other biophysical effects that are found in brain tumours.

VERDICT-MRI (Vascular, Extracellular, and Restricted DIffusion for Cytometry in Tumours) is an imaging and computational modelling framework for characterising the vascular, extracellular, and restricted components of tumours [34]. It has shown diagnostic utility and high repeatability in body tumours, especially prostate cancer [35,36], but also metastases [37]. The application of VERDICT in brain tumours is particularly challenging because of the brain’s complex tissue microstructure and cancer heterogeneity. Even though the cores of most brain lesions are microstructurally similar to body tumours, areas of infiltration and peritumoural oedema may be anisotropic or present other features that may not be adequately modelled by VERDICT in its formulation tailored to body tumours.

The aim of this study was to develop an appropriate microstructural model for brain tumours using the VERDICT framework. We used an extensive acquisition protocol, which necessarily limited the population of this preliminary study. Given the exploratory nature of this study, we chose not to focus on a specific type of brain tumour, but rather to have a diverse population, prioritising the aim of testing our framework in most conditions that can be realistically expected in patients with brain tumours over a thorough statistical analysis in a smaller subgroup. We focused on exploring clinically relevant features using VERDICT estimates: cell density and size, vascularity, infiltration, and oedemas. We validated VERDICT estimates against histopathology both in cases with whole tumour surgical resection and in cases that underwent stereotactic biopsies, with the latter allowing more precise and localised comparisons in heterogeneous lesions. We also compared the vascularity estimates with independent measures from perfusion MRI, such as plasma volume or cerebral blood volume.

## 2. Materials and Methods

### 2.1. Subjects

Data were collected from 21 patients admitted to the Department of Neurosurgery and Gamma Knife Radiosurgery of San Raffaele Hospital (Milan, Italy) harbouring intracerebral lesions and suspected for either a primary or secondary brain tumour. The inclusion criteria were as follows: suspected diagnosis of brain tumour based on previously acquired brain contrast-enhanced MRI, adult patient, patient eligible for either surgical resection or stereotactic needle biopsy (depending on tumour location/extension, suspected diagnosis, and patient characteristics such as age, comorbidities, and performance status), and patient consent to undergo an extensive advanced MRI acquisition protocol and use of their data, anonymously, for scientific studies. The exclusion criteria were: paediatric age, presence of diagnostic hypotheses other than that of brain tumour, absence of indication for surgical resection or biopsy, and non-cooperation from a patient or unwillingness to provide consent for study recruitment.

The patient cohort included 21 adult patients with suspected brain tumours (16 M, 5 F; mean age, 52 years; range, 19–77 years). Among them, 16 patients had gross tumour resection (GTR) and 5 underwent stereotactic biopsy.

### 2.2. MRI Acquisition

MRI was performed preoperatively with a 3.0T Ingenia CX scanner (Philips Healthcare, Best, The Netherlands) at the Neuroradiology Unit and CERMAC, IRCCS Ospedale San Raffaele (Milan, Italy). Conventional MRI including 3D-FLAIR images (TE/TI/TR = 285/2500/9000 ms, isotropic resolution 0.7 mm) and post-contrast 3D T1-weighted images (TE/TR = 5.27/11.12 ms, flip angle 8°, isotropic resolution 0.5 mm) were acquired.

dMRI scans were acquired using the parameters summarised in Table 1 and with an isotropic voxel size of 2 mm. Nineteen of the patients also had perfusion MRI, including dynamic contrast-enhanced (DCE) 3D spoiled gradient echo sequences (TE/TR = 1.8/3.9 ms, flip angle 15°, in-plane resolution 2 × 2 mm^2^, slice thickness 2.5 mm, 70 repetitions) and dynamic susceptibility contrast (DSC) fast field echo EPI sequences (TE/TR = 31/1500 ms, flip angle 75°, in-plane resolution 2 × 2 mm^2^, slice thickness 5 mm, 80 repetitions).

### 2.3. Processing of Diffusion and Perfusion MRI

We performed denoising and removal of Gibbs artifacts on the dMRI data using MRtrix3 [38,39,40] and motion and distortion correction using MD-dMRI [41,42].

Perfusion MRI data were analysed using Olea Medical software (v3.0, Olea Medical Solutions, La Ciotat, France) to obtain parametric maps of Plasma Volume (Vp) and Relative Blood Volume (rBV). These maps were registered to the first b = 0 volume of the dMRI data using the FMRIB’s Linear Image Registration Tool (FLIRT) [43] from the FMRIB Software Library (FSL v 6.0, FMRIB, Oxford, UK).

Regions Of Interest (ROI) masks were segmented on 3D-FLAIR images for the whole tumour. In patients with contrast-enhancing tumours, segmentation of the tumour core was performed on the post-contrast 3D-T1 images, whereas in patients with non-enhancing tumours, the 3D-FLAIR images were used. Cystic or necrotic regions were excluded from the segmentation. Both anatomical images were registered to the first b = 0 volume using FLIRT, and the periphery mask was obtained as the difference between the whole tumour and tumour core in contrast-enhancing tumours. To minimise misregistration issues, both core and periphery masks were eroded, visually checked in all patients, and manually corrected where necessary, especially in heterogeneous lesions.

### 2.4. Diffusion Models of Tissue

According to the VERDICT framework, we created different three-compartment models to characterise the vascular, intracellular, and extracellular components of brain tumours. Using the terminology in [22], we used Sphere to model intracellular restriction in tumour cells, as in [34,44]; we considered Zeppelin, Tensor, Cylinder, Stick, and Watson-distributed Sticks for the extracellular compartment, accounting for the extracellular space and possibly white matter fibres and/or areas of oedema; and we used Ball and AstroSticks for the vascular compartment. The name of each multi-compartment model was formed first by the extracellular compartment, then the vascular compartment, and finally the restricted compartment. We fit the models to the data using custom code in Matlab (MathWorks, Natick, MA, USA) based on the fmincon function for constrained optimisation using the active set algorithm. The parameters of each compartment model and the constraints we applied to each of them are reported in Supplementary Table S1. In particular, we constrained the Pseudo-Diffusivity (dv) to be ≥3 × 10^−9^ m^2^/s when using Ball or ≥9 × 10^−9^ m^2^/s when using AstroSticks; we also evaluated models with dv fixed to 1.5 × 10^−8^ m^2^/s (Ball) or 4 × 10^−8^ m^2^/s (AstroSticks) based on exploratory results. We assumed that all of the compartments shared the same T2, there was no exchange between them, and compartment-specific diffusivities were not time-dependent.

To account for areas of oedema, necrosis, and cysts that are often seen in brain tumours, we tested a Free Water Elimination (FWE) procedure based on fitting the NODDI model to the data in the last two shells (see Table 1). Our FWE approach consisted of adding a Ball compartment to the model, with the diffusivity fixed to 3 × 10^−9^ m^2^/s and the Signal Fraction (ffw) fixed to the value estimated by NODDI for the isotropic compartment (fiso). This effectively resulted in four-compartment models with the same number of free parameters as the corresponding three-compartment models with no FWE.

We also fitted the standard monoexponential model on the b = 1000 s/mm^2^ shell to calculate the ADC as a clinical standard reference.

### 2.5. Selection of the VERDICT Mathematical Model for Brain Tumours

Starting from all the possible combinations of compartments listed in the previous section, we followed a cascade model selection procedure including the following evaluations:

1. To select the best extracellular compartment model without the confounding effect of a low-b dMRI signal that is mainly related to pseudo diffusion, we used the Corrected Akaike’s Information Criterion (AICc) to evaluate the fitting performance of the two- and three-compartment models on high-b data (b > 200 s/mm^2^, excluding the first 4 shells in Table 1);
2. To assess the fitting performance on the full signal, we evaluated the AICc again on the best-performing models from (1) with the addition of the vascular compartment (Ball or AstroSticks), and with and without FWE;
3. We evaluated anisotropic measures in the extracellular compartment of the same models as in (2), fitted to the full signal. ODI from NODDI was considered as the gold standard;
4. To highlight issues of ambiguity between pseudo-diffusion and diffusion with high diffusivity, we investigated estimates of the Vascular Fraction (fvasc) from the same models as in (2), fitted to the full signal, in areas where NODDI provided fiso >0.5 and we did not expect any significant vascularity.

### 2.6. Histology

Tissue samples were immediately fixed in 10% formalin solution and sent to the Pathology Department, where they were processed the same day or the day after in cases in which the procedures were performed in the late afternoon. Histopathological diagnosis was performed according to the 2016 WHO classification of CNS tumours [45]. In cases of stereotactic biopsy, the procedure began with fixation of the patient’s skull in the MRI-compatible Leksell stereotactic frame (Model G, Elekta, Stockholm, Sweden). During the procedure, a Sedan biopsy needle (10 mm needle window, 2.5 mm diameter) was used to acquire at least two cylindrical tissue biopsies, based on the macroscopical aspect, size of the sample, and procedure-related risks. The bioptic sampling accuracy was eventually confirmed via co-registering the postoperative CT images with preoperative imaging and the planned trajectory in the Leksell SurgiPlan^®^ (Elekta Instruments AB, Stockholm, Sweden) software for all cases in the series. The stereotactic coordinates of the exact, final sites of biopsy were exported from the Surgiplan software and transferred to VERDICT maps using the transformations obtained when registering the T1-weighted images to dMRIs.

### 2.7. Statistical Analysis

To assess clinical utility, we evaluated the parameters from the model that performed best according to our model selection procedure, and we calculated descriptive statistics in tumour cores and periphery ROIs, then comparing the median values between different histotypes. For this purpose, we first evaluated the normality of data distributions using the Shapiro–Wilk test, then we applied one-way ANOVA with Tukey’s multiple comparisons to normal data and the Kruskal–Wallis test with Dunn multiple pairwise comparisons to non-normally distributed data, with a significance level of *p* < 0.05 in both cases. We also evaluated the relationship between the vascular fraction from VERDICT and well-assessed PWI-derived metrics Vp and rBV with a similar physiological background. For this task, we calculated the Pearson correlation coefficient (r) between fvasc and Vp or rBV.

## 3. Results

### 3.1. Patient Population

The patients’ histopathological and molecular data are summarised in Table 2; more details are reported in Supplementary Table S2.

Histopathological analysis resulted in 10/21 patients having a lower grade astrocytoma (4 WHO 2, 6 WHO 3) and 5/21 patients having a glioblastoma WHO 4. Two patients had ependymal tumours (1 subependymoma WHO 1, 1 ependymoma WHO 3), two had brain metastases from melanoma, and one had pathology-proven radionecrosis after previous radiosurgical treatment of a solitary brain metastasis from lung adenocarcinoma. One had a focal cortical dysplasia, resembling a low-grade glial neoplasm on preoperative imaging.

**Figure 1:**
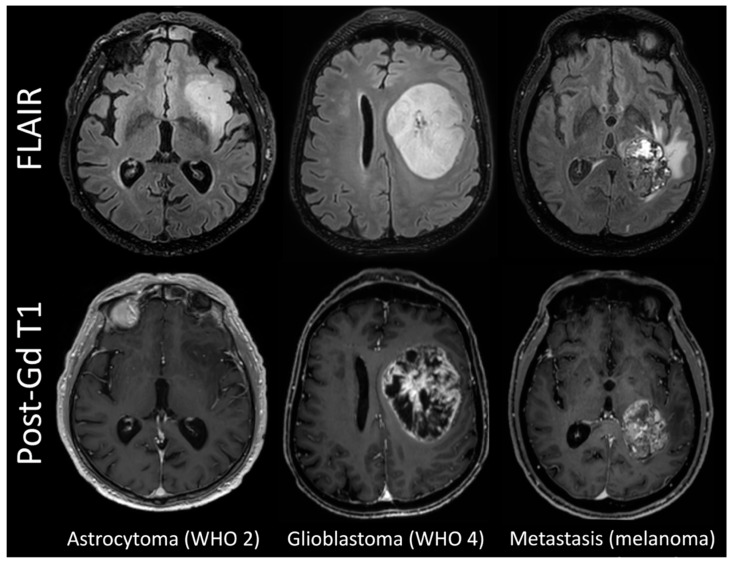
FLAIR and post-contrast T1w images in representative cases.

The WHO 2 gliomas tended to be more homogeneous on FLAIR images and have no contrast enhancement on T1-weighted images, whereas most WHO 3 gliomas showed discrete foci of enhancement; glioblastomas and metastases presented as large lesions with central heterogeneous enhancement surrounding necrosis and peripheral oedema on FLAIR images (Figure 1).

For quantitative analysis of the VERDICT parameters in the tumour core, lesions were classified according to different WHO grades and histologies.

### 3.2. Selection of the VERDICT Mathematical Model for Brain Tumours

**Figure 2:**
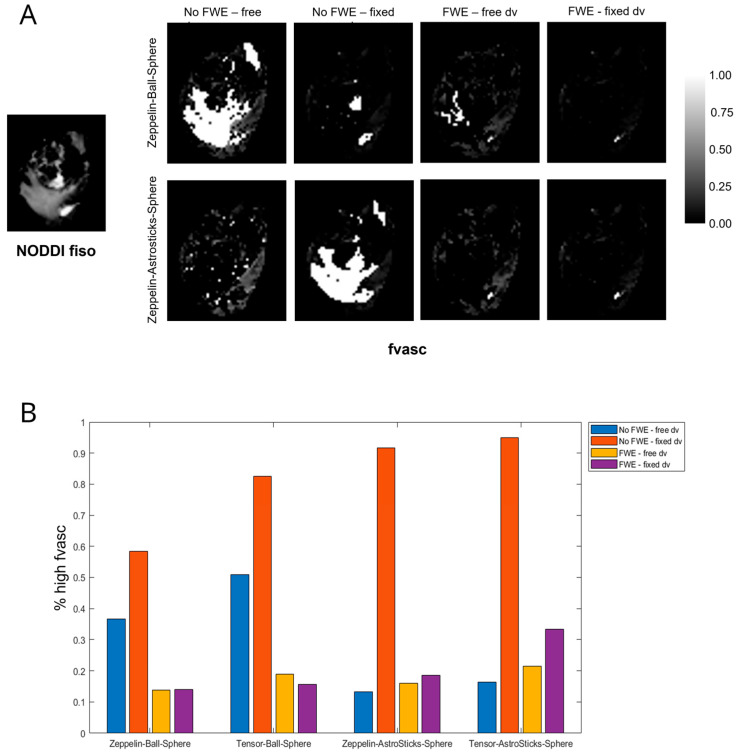
Spurious high fvasc in areas with high water content and effect of FWE and fixing dv. (**A**) An example where very high fvasc values were estimated by some of the considered models in an area of oedema with high water content (see the free water fraction estimated by NODDI on the left). (**B**) Bar graph showing the percentage of voxels with fvasc >0.9 out of those with fiso >0.5, averaged among all patients, for the four considered VERDICT models.

Following the cascade procedure described in the Materials and Methods (Section 2.4), we found that:

1. In the comparison between two- and three-compartment models at high b, all of the top-performing (lower AICs) models included Tensor and/or Zeppelin to describe the extracellular and extravascular compartments, whereas models including Stick, Watson-distributed Sticks, or Cylinder to describe the extracellular and extravascular compartments performed worse on average. For this reason, only Tensor and Zeppelin were considered as candidates for the extracellular compartment in the following experiments. The considered models are ranked according to the average AICc across patients in Supplementary Table S3;
2. In all cases, the AICc of the model with FWE was significantly lower (better fitting) or very similar than that without FWE. The difference was higher in peritumoural areas and when the pseudo-diffusivity was fixed in the vascular compartment. The average AICc values of the considered models, fitted to the full signal, are reported in Table 3;
3. Comparing equivalent models in which the only difference was the form of the extracellular compartment (Zeppelin or Tensor), models with Zeppelin showed stronger correlations with NODDI. The correlation coefficients between the ROI-averaged FA of the extracellular compartment from each model and the ROI-averaged ODI from NODDI are listed in Table 4;
4. Very high values of fvasc were often estimated in areas of high fiso in models without FWE and when using Ball (as opposed to AstroSticks) to model the vascular compartment. We assumed that such high values were biased and symptomatic of model degeneracy, as vascularity should be negligible in extracellular areas with high free water content; an example is shown in Figure 2A. To quantitatively assess this observation, we measured the percentage of voxels with fvasc >0.9 for each model out of those with fiso >0.5 in NODDI. The highest values were found for models with fixed diffusivity of the vascular compartment without FWE; FWE reduced the extent of this issue especially when dv was fixed and the AstroSticks model seemed to be more robust (Figure 2B).

Based on these results, we selected the Zeppelin–AstroSticks–Sphere model with FWE and dv fixed to 4 × 10^−8^ m^2^/s.

### 3.3. Comparison between Histotypes

**Figure 3:**
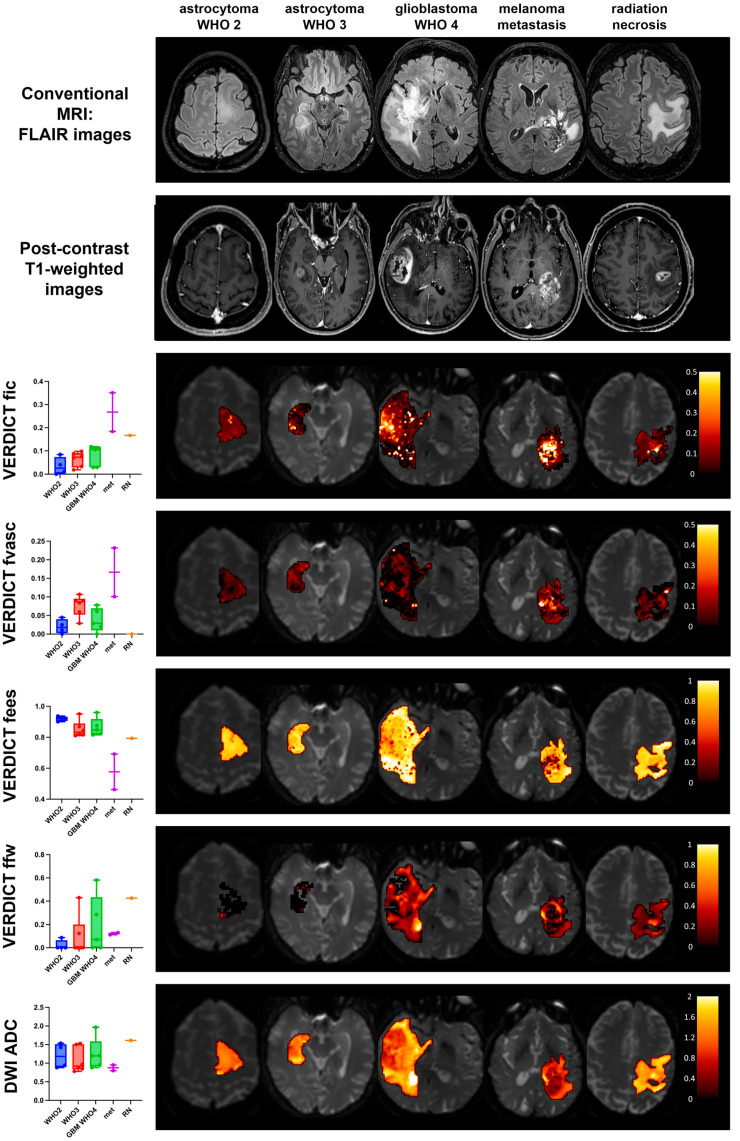
VERDICT and ADC results in the tumour cores. The boxplots on the left show the median values in each patient group for the signal fraction of each compartment in the tumour core (i.e., the enhancing tissue on post-contrast T1 images or FLAIR abnormalities in non-enhancing tumours). Beside each plot, representative maps are shown for each group. The VERDICT maps are colour-coded and overlayed on b = 0 images showing parameter values in the whole tumour area, including both the lesion core and peritumoural area on the selected slice (i.e., the whole FLAIR abnormality).

Boxplots of VERDICT signal fractions in tumour cores for the different histotypes and grades are shown in Figure 3 along with maps from representative cases; the ADC is also included as a clinical standard reference. Based on a qualitative evaluation, areas of increased Intracellular Fraction (fic) in VERDICT maps tended to be larger in tumours with high cellularity (glioblastoma and metastasis). Quantitative analysis showed a trend toward higher values of Intracellular Fraction (fic) within tumour cores with increasing grade; fic values were also significantly higher in metastases than in lower grade gliomas (see Supplementary Figure S1, met vs. WHO 2: *p* = 0.05). The Extracellular Fraction (fees) values were higher in WHO 2 than in WHO 3 and GBM, although not significantly (Figure 3); metastases showed significantly lower fees values than WHO 2 gliomas (see Supplementary Figure S1, *p* = 0.03). There was a trend towards decreasing Free Water Fraction (ffw) going from GBM to WHO 3 and WHO 2; however, none of these differences were statistically significant. The radionecrosis case had very high free water content and no vascular fraction, as expected. By contrast, the ADC quantitative values were similar across the different histotypes, demonstrated by the large overlap of the ADC boxplots shown in Figure 3.

Benign histotypes such as subependymoma WHO 1 and focal cortical dysplasia (Supplementary Figure S1) had low fic values and high fees with low ffw and fvasc, as expected. Anaplastic ependymoma WHO 3 showed relatively high values of fic and low values of fees in the tumour core, consistent with the histological findings of high nuclear to cytoplasmic ratios and high mitotic activity with relatively low free water content and vascular fraction, consistent with the scarce microvascular proliferation and necrosis.

**Figure 4:**
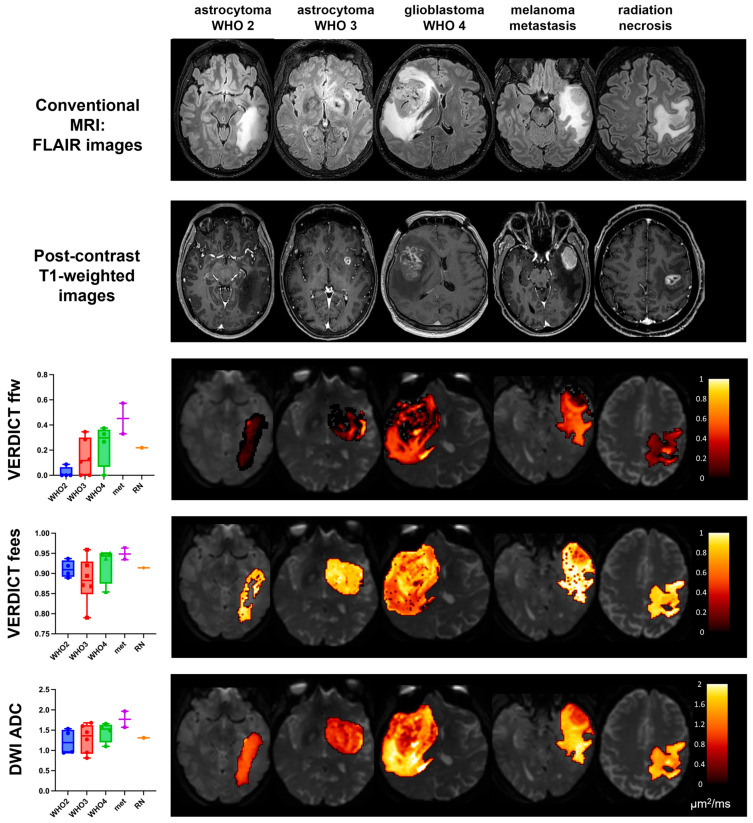
VERDICT and ADC results in the peritumoural areas. The boxplots on the left show the median values in each patient group for the signal fraction of each compartment in the peritumoural area (i.e., the difference mask between whole tumours and tumour cores in contrast-enhancing tumours, and the whole FLAIR mask in non-enhancing tumours). Beside each plot, representative maps are shown for each group. The VERDICT maps are colour-coded and overlayed on b = 0 images showing parameter values in the whole tumour area (i.e., the whole FLAIR abnormality).

Boxplots of the Free Water Fraction (ffw), Extracellular Fraction (fees), and ADC in peritumoural areas are shown in Figure 4 for the different histotypes along with maps from representative cases. Vasogenic oedemas around metastases had significantly higher ffw values than the periphery of WHO 2 lesions (*p* = 0.035) and tended to have larger ffw values than infiltrative oedemas (around WHO 3 gliomas and GBM), and infiltrative oedemas tended to have larger ffw values than the periphery of WHO 2 lesions. The ADC also showed an increasing trend from the periphery of WHO 2 lesions to infiltrative oedemas around WHO 3 lesions to vasogenic oedemas around metastases, although showing largely overlapping values.

No significant differences in VERDICT-derived parameters were found between IDH-mutant and wild-type gliomas, although fic values tended to be higher in the latter, as expected (Supplementary Figure S2).

### 3.4. Comparison between VERDICT Fvasc and PWI Parameters

**Figure 5:**
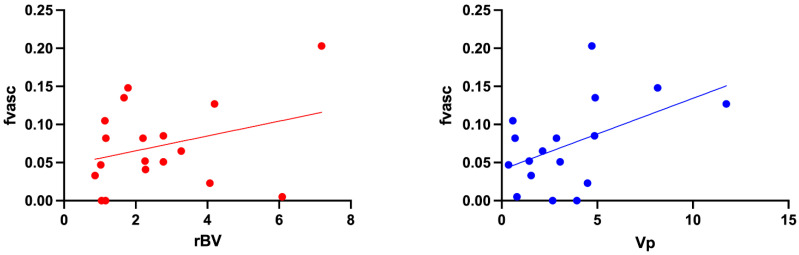
Correlations between VERDICT fvasc and PWI metrics in the tumour core; correlation with rBV (from DSC data) is shown on the left, correlation with Vp (from DCE data) is shown on the right.

In Figure 5, fvasc values from VERDICT are plotted against PWI-derived parameters rBV and Vp in the core of each lesion. The Pearson correlation test revealed a statistically significant, positive correlation between fvasc and Vp values (r = 0.489, *p* = 0.046), but not between fvasc and rBV values (r = 0.313, *p* = 0.206).

### 3.5. Comparison between Histology and VERDICT Maps

**Figure 6:**
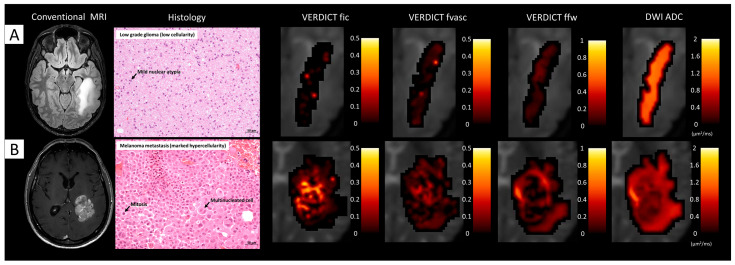
Comparison between VERDICT maps and histology from gross resection. (**A**) The first case (Patient 19) underwent surgical resection for a large left temporo-parietal low-grade astrocytoma (WHO 2, IDH-mutated); the histopathological section shows mild cellularity with the presence of fibrillary neoplastic astrocytes on a loose tumour matrix background; some mild nuclear atypias are identifiable. (**B**) The second case (Patient 4) underwent surgical removal for a large melanoma metastasis; histopathology shows marked hypercellularity with epithelioid cells with abundant cytoplasm, large nuclei, and prominent nucleoli interlaced with extravasated red blood cells.

Figure 6 shows VERDICT and ADC maps and histological images from gross resection of a low-grade glioma and a metastasis. In the former case, VERDICT showed very low fic and fvasc values, compatible with the low cellularity shown by histology. In the latter case, VERDICT estimated areas of very high fic and fvasc in the core of the tumour, in agreement with the high cellularity that is typical of malignant tumours such as metastatic melanoma. The free water fraction was high in the peritumoural area (vasogenic oedema) and in some intratumoural spots that may have corresponded to necrosis. ADC maps qualitatively showed less heterogeneity across the tumour tissues.

**Figure 7:**
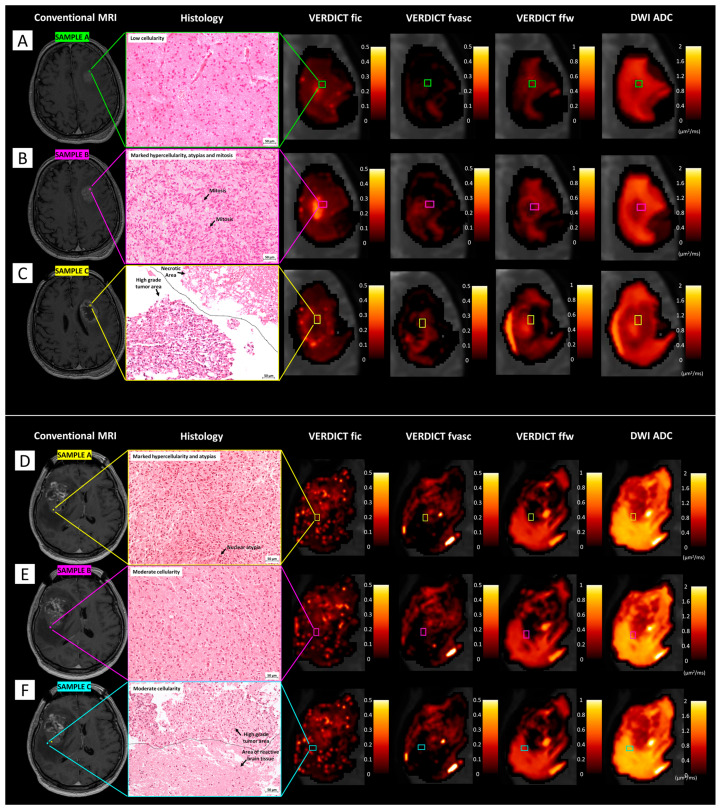
Comparison between VERDICT maps and histology from stereotactic biopsy. (**A**–**C**) The first case (patient 12) underwent stereotactic biopsy for a left frontal WHO 4 glioblastoma (IDH-1 wild-type). From a histopathological point of view, the first sample (**A**) was characterised by mild cellularity; the second bioptic sample (**B**) was performed in a higher grade tumour area, characterised by marker hypercellularity, atypias, and mitotic figures; in the third sample (**C**), both necrotic and high grade tumour areas were identified. Note the increase of fic values from periphery (**A**) to the tumour ‘vital’ core (**B**) and the drop in fic values and marked increase in ffw values in the necrotic core (**C**). (**D**–**F**) The second case (patient 3) underwent stereotactic biopsy for a right frontal WHO 4 glioblastoma (IDH-1 wild-type). Histopathologically, the first sample (**D**) was characterised by marker hypercellularity and atypias; the second sample (**E**) entailed areas of moderate cellularity; the last sample (**F**) showed areas of high-grade tumour adjacent to areas of reactive brain tissue with possible infiltration. Note the progressive reduction in fic values from (**D**) to (**F**), along with the increase in ffw values, possibly due to oedema.

Figure 7 shows VERDICT and ADC maps and histological images in multiple samples from stereotactic biopsies in two patients harbouring high-grade gliomas. In the first case (patient 12), fic values were higher in the area corresponding to sample B, with histological features of high-grade glioma, than in the area corresponding to sample A, with histopatological features of infiltrating glioma cells. In the area corresponding to sample C, fic values were very low and ffw values were the highest, corresponding to necrosis as shown by histology. In the second case (patient 3), fic values were higher in sample A than in the other two samples, compatible with the higher grade shown by histology; fvasc values were also slightly higher in sample A. In contrast, ffw values were low in sample A, intermediate in sample B, and highest in sample C, compatible with the presence of oedema.

## 4. Discussion

This study developed and validated a microstructural model for brain tumours within the VERDICT framework. We focused on deriving estimates characterising clinically relevant features such as cell density, vascularity, infiltration, and oedemas. We validated the VERDICT non-invasive estimates against histopathology from stereotactic biopsies in different types of tumours and peritumoural areas and independent measures, such as plasma volume or cerebral blood volume from perfusion MRI.

We examined numerous diffusion models for the three VERDICT components. Following model selection, the best-performing model was the Zeppelin–AstroSticks–Sphere model with free water elimination. To model the extracellular space, Zeppelin performed best, and we showed that it could characterise anisotropy in and around the lesions similarly to NODDI. As in VERDICT for body tumours [35], AstroSticks was preferred to Ball for the vascular compartment. In particular, the Ball model tended to estimate high values of the vascular fraction in areas of significant free water content such as oedemas, where we did not expect any vascularity, meaning that such a model for vasculature could not reliably distinguish true diffusion with high diffusivity from pseudo-diffusion in vessels. Using AstroSticks rather than Ball in the vascular compartment helped to reduce this degeneracy. To further address this phenomenon, we included a free water compartment to give the models enough flexibility to capture all of the different aspects of the complex microstructure of brain tissue. Without increasing model complexity, we achieved this by using a two-stage free water elimination approach; we fitted NODDI to the signal at higher b values to estimate the free water fraction, which was then fixed in the subsequent fitting of the full VERDICT model. In this way, we effectively obtained four-compartment models without extra free parameters, and we showed that this improved the fitting performance, especially in peritumoural areas.

VERDICT maps reflected the main features shown by histology in low-grade cases and in the most aggressive metastases, and even differences between multiple biopsy samples in the same patients. The qualitative comparison shown here holds great promise for the non-invasive characterisation of brain tumours, as VERDICT could provide more specific microstructural information not only about the entirety of each lesion, but also about the different areas of heterogeneous lesions with respect to standard clinical ADC maps. This would allow the identification of the most aggressive components, which would be fundamental in the context of surgery or treatment planning. One limiting factor for the correlation was that the biopsies were not planned specifically for this study and were only available for a few of our patients; furthermore, uncertainty in the localisation of biopsy samples on morphological images and in the registration to dMRI images limited the spatial accuracy of the comparisons. More systematic correlation studies will be carried out in the future, with localised quantification of histopathology.

The preliminary analysis of the distribution of quantitative values derived from VERDICT maps in different histotypes showed that melanoma metastases had the highest intracellular and vascular fractions and the lowest extracellular fraction in the core, which we expected as these lesions are known to have particularly dense cell packing and high vascularity. Higher grade tumours also tended to have higher intracellular and vascular fractions and lower extracellular fractions than lower grades, as expected. In the radionecrosis case, the free water fraction was very high, showing that our VERDICT model did not provide spurious cellularity or vascularity where no residual tissue was present. Although metastases and radionecroses are clearly very different from gliomas, they were included in this exploratory study to evaluate the sensitivity of the proposed approach in a wide range of microenvironments. A large overlap of values was found in the free water fraction between the different histopathological entities; as we focused on the solid component of lesions, larger areas of necrosis and cysts were not considered in the analysis, so only minor differences could be expected between grades. In the peritumoural areas, we observed a trend towards a higher free water fraction in purely vasogenic oedemas compared to that in infiltrative oedemas.

Despite the small sample size that does not allow generalisations on the statistical significance of the results, the patient population of this study was quite diverse, including the most common histopathological and molecular subtypes of intra-axial brain tumours and different pathological features even within the same group, allowing us to test VERDICT in a wide spectrum of conditions. Furthermore, the potential usefulness of the metrics derived from the VERDICT analysis was shown against the substantial overlap of ADC values, which are commonly used in the clinic. Finally, the greater heterogeneity of the tumour area in VERDICT maps with respect to that in ADC maps suggests the potential to define areas of different aggressiveness in the context of the same lesion as well as biologically ‘inert’ areas such as radionecrosis. Future studies focusing on a few classes, but with a larger population, would be needed to assess the significance of VERDICT parameters to differentiate brain tumour histotypes as well as to assess the validity of the model to differentiate other molecular subtypes (i.e., 1p19q codeletion in lower-grade gliomas) or in extra-axial tumours such as meningiomas. We must also highlight that in this preliminary evaluation we only compared ROI-averaged values, neglecting within-lesion variability in the comparison between tumour types; nonetheless, the observed trends reflected the expected underlying microstructure. Assessing these differences would be clinically relevant, as it would allow the non-invasive characterisation of tumour heterogeneity and microstructure, giving clinicians very valuable information to plan surgeries, bioptic procedures, or to monitor the effect of therapies.

Comparing the vascular VERDICT estimate fvasc with independent perfusion MRI metrics, we found a certain degree of correlation between the VERDICT vascular fraction and PWI-derived rBV and Vp, but the correlation coefficient was not as high as expected. We speculate that this discrepancy may be due to the different mechanisms underlying PWI models and IVIM [31,46,47]. Additionally, the PWI and dMRI images had different spatial resolutions and we can expect some degree of misregistration between rBV/vp and fvasc maps, which might have contributed to the decreased correlation.

The VERDICT formulation for body cancer has been applied to brain tumours, showing some differentiation between glioma types in patients and response to therapy in a mouse model [48,49]. However, using the microstructure model developed for body tumours may lead to biased estimates of the parameters and make the interpretation of results more difficult because the model cannot adequately capture the complexity of brain tissue. In particular, our proposed VERDICT model can account for anisotropy and separate the contribution of free water and brain tissues, which is particularly important in peritumoural areas.

In addition to the relatively small and heterogeneous population, as discussed above, the main limitation of the current study was the long time needed for both acquisition and image processing, which would not be acceptable in a clinical context and was a limiting factor for the study population. The rich acquisition protocol was designed to allow us to fit an extended range of multi-compartment models to identify the appropriate VERDICT model for brain tumour tissue, but it is still too time-consuming to be included in clinical routines or even in large research studies. Having identified the optimal model, we can now optimise the acquisition protocol using the procedure in [50]. We can expect that the number of diffusion directions may be reduced, as we found that Zeppelin was sufficient to characterise the extracellular anisotropy and more complex models are unnecessary. The next step is, therefore, to design a clinically feasible protocol (approximately 10 min) using an optimisation technique such as that described in [50,51] to shorten the acquisition time without compromising the diagnostic utility of the derived VERDICT biomarkers. The long time required for fitting was mainly due to our conservative choice of using the traditional non-linear least squares optimisation. In the future we will adopt machine-learning approaches, which will dramatically reduce the computational time and may also help to avoid local minima and regularise the parameter maps [44,52,53]. Machine learning could also be used for free water elimination [54].

From a modelling point of view, we did not account for relaxation bias, time-dependence of compartmental diffusivities, and/or exchange between compartments. Since we assumed the same T2 for all compartments and the acquisition protocol included a wide range of echo times, our estimated tissue fractions may be biased by T2 differences between the intracellular, extracellular, and vascular spaces. Future studies will investigate the possibility of estimating a different T2 value for each compartment; some constraints on other parameters may be needed to avoid increasing the model’s complexity. Estimates of VERDICT metrics may also have been biased by the time dependence of diffusivity parameters, which has recently been shown to be significant in the GL261 mouse model for diffusion times up to 150 ms; on the other hand, exchange should have little effect at the considered diffusion times [55].

## 5. Conclusions

In conclusion, we optimised the VERDICT framework for application to brain tumours, aimed at simultaneously and non-invasively characterising cellular and vascular features both in the core of the lesion and in the surrounding tissue. Our model proved to be flexible, robust, and gave promising preliminary results in agreement with histology, showing its potential for improving the management of brain tumour patients. The next steps will focus on optimising the method for clinical standards with a shorter protocol and applying it to larger patient cohorts.

## Figures and Tables

**Table 1:** Acquisition parameters for the dMRI protocol. Abbreviations: b = b-value (degree of diffusion weighting), TE = echo time, δ = diffusion gradient duration, Δ = diffusion gradient separation, Ndir = number of diffusion gradient directions.

**b (s/mm^2^)**	50	70	90	110	350	1000	1500	2500	3000	3500	711	3000
**TE (ms)**	45	53	43	43	54	78	118	88	103	123	78	78
**δ (ms)**	5	5	5	5	10	10	10	20	15	15	20	20
**Δ (ms)**	22	30	20	20	26	50	90	50	70	90	42	42
**Ndir**	3	3	3	3	3	3	3	3	3	3	38	63

**Table 2:** Summary of patient sample characteristics.

**Age**	mean 52 years (range 19–77 years)
**Sex**	
Male	16
Female	5
**Histopathology**	
*** Glioma***	**17**
Lower grade glioma (WHO 2-3)	10
* IDH1/2 mutated* ^a^	*4 (1 WHO 2, 3 WHO 3)*
* IDH1/2 wild-type* ^a^	*6 (3 WHO 2, 3 WHO 3)*
Glioblastoma (WHO 4)	5
* IDH1/2 wild-type*	*5*
Other glial tumours (ependymal) ^a^	2 (1 subependymoma WHO 1, 1 ependymoma WHO 3)
*** Metastasis***	**2** (melanoma)
*** Other***	**1** radiation necrosis, **1** focal cortical dysplasia

^a^ number of lesions for each WHO grade are reported in parentheses.

**Table 3:** AICc of the considered models with and without FWE, averaged across patients. The lower value of each pair is highlighted in bold if the difference is significant according to the Wilcoxon signed-rank test.

Model	Core AICc		Periphery AICc	
	No FWE	FWE	No FWE	FWE
Zeppelin–Ball–Sphere	2538	2541	2595	**2582**
Zeppelin–Ball–Sphere with fixed dv	2538	2545	2583	2582
Tensor–Ball–Sphere	2536	2546	2606	2606
Tensor–Ball–Sphere with fixed dv	2546	**2543**	2632	**2571**
Zeppelin–AstroSticks–Sphere	2542	2543	2583	2583
Zeppelin–AstroSticks–Sphere with fixed dv	2616	**2553**	3436	**2598**
Tensor–AstroSticks–Sphere	2541	2541	2572	2574
Tensor–AstroSticks–Sphere with fixed dv	2668	**2641**	5993	**2726**
Zeppelin–Ball –Sphere	2538	2541	2595	**2582**

**Table 4:** Pearson correlation coefficients between the mean FA of the extracellular compartment of each model and the mean ODI from NODDI in the same area (core or periphery). For each pair of models (equivalent except for the extracellular compartment, which is Zeppelin on one side and Tensor on the other), the stronger correlation is highlighted in bold.

Model (Excluding Extracellular Compartment)	Core		Periphery	
	Zeppelin	Tensor	Zeppelin	Tensor
Ball–Sphere	**−0.63**	−0.49	+0.13	+0.09
Ball–Sphere with fixed dv	**−0.50**	−0.48	**−0.19**	−0.04
Ball–Sphere with FWE	**−0.65**	−0.44	**−0.35**	−0.11
Ball–Sphere with FWE and fixed dv	**−0.46**	−0.45	−0.39	**−0.42**
AstroSticks–Sphere	**−0.62**	−0.45	**−0.19**	−0.05
AstroSticks–Sphere with fixed dv	**−0.29**	−0.27	+0.13	+0.13
AstroSticks–Sphere with FWE	**−0.65**	−0.42	**−0.36**	−0.32
AstroSticks–Sphere with FWE and fixed dv	**−0.42**	−0.16	**−0.42**	−0.16
Ball–Sphere	**−0.63**	−0.49	+0.13	+0.09

## Data Availability

The datasets for this study are available from the authors upon request.

## Supplementary Materials

The following supporting information can be downloaded at: www.mdpi.com/article/10.3390/cancers15092490/s1. Table S1: List of the considered compartment models with the associated parameters and constraints. Table S2: Details of patients’ characteristics. Table S3: Ranking of two- and three-compartment models fitted on high-b signals, based on the average AICc across patients. A single-Ball model is also included for comparison. Figure S1. VERDICT results in the tumour core for the whole cohort of patients. The boxplots show the ROI median values in each patients group for the signal fraction of each compartment. Statistically significant differences are marked (* *p* < 0.05). Figure S2. VERDICT results in the tumour core according to IDH status. The boxplots show the ROI median values in each patients group for the signal fraction of each compartment.
