# Supplementary material for: Comprehensive Brain Tumour Characterisation with VERDICT-MRI: Evaluation of Cellular and Vascular Measures Validated by Histology
Source: Cancers (Basel). 2023 Apr 27;15(9):2490. doi: 10.3390/cancers15092490 (PMC10177485; doi:10.3390/cancers15092490)
Supplement: Supplementary file 1 [file cancers-15-02490-s001.zip › cancers-2280062-supplementary.pdf]

cancers

MDPI

*Supplementary Materials*

# Comprehensive brain tumour characterization with VERDICT-MRI: evaluation of cellular and vascular measures validated by histology

**Supplementary Table S1**: List of the considered compartment models with the associated parameters and constraints.

| Compartment | Model | Parameters | Constraints |
|---|---|---|---|
| **Extracellular** (fraction **fees**) | **Zeppelin** | Main orientation: θ, ϕ | None |
| | | Diffusivity along the main axis: $D_{/\!/}$ | $1{\cdot}10^{-11}$ m²/s ≤ $D_{/\!/}$ ≤ $3{\cdot}10^{-9}$ m²/s |
| | | Diffusivity in the orthogonal plane: $D_{\perp}$ | $1{\cdot}10^{-11}$ m²/s ≤ $D_{\perp}$ ≤ $3{\cdot}10^{-9}$ m²/s<br>$D_{\perp} \leq D_{/\!/}$ |
| | **Tensor** | Main orientation: θ, ϕ | None |
| | | Diffusivity along the main axis: $D_{/\!/}$ | $1{\cdot}10^{-11}$ m²/s ≤ $D_{/\!/}$ ≤ $3{\cdot}10^{-9}$ m²/s |
| | | Diffusivity in the 1st orthogonal direction: $D_{\perp 1}$ | $1{\cdot}10^{-11}$ m²/s ≤ $D_{\perp 1}$ ≤ $3{\cdot}10^{-9}$ m²/s<br>$D_{\perp 1} \leq D_{/\!/}$ |
| | | Diffusivity in the 2nd orthogonal direction: $D_{\perp 2}$ | $1{\cdot}10^{-11}$ m²/s ≤ $D_{\perp 2}$ ≤ $3{\cdot}10^{-9}$ m²/s<br>$D_{\perp 2} \leq D_{\perp 1}$ |
| | **Stick** | Main orientation: θ, ϕ | None |
| | | Diffusivity along the main axis: $D_{/\!/}$ | $1{\cdot}10^{-11}$ m²/s ≤ $D_{/\!/}$ ≤ $3{\cdot}10^{-9}$ m²/s |
| | **WatsonSticks** | Main orientation: θ, ϕ | None |
| | | Intrinsic diffusivity: Dees | $1{\cdot}10^{-11}$ m²/s ≤ Dees ≤ $3{\cdot}10^{-9}$ m²/s |
| | | Orientation dispersion: ODI | None |
| | **Cylinder** | Main orientation: θ, ϕ | None |
| | | Intrinsic diffusivity: Dees | $1{\cdot}10^{-11}$ m²/s ≤ Dees ≤ $3{\cdot}10^{-9}$ m²/s |
| | | Radius: $R_{cyl}$ | $1{\cdot}10^{-7}$ m ≤ $R_{cyl}$ ≤ $2{\cdot}10^{-5}$ m |
| **Vascular** (fraction **fvasc**) | **Ball** | Pseudo-diffusivity: dv | $3{\cdot}10^{-9}$ m²/s ≤ dv ≤ $1{\cdot}10^{-7}$ m²/s<br>or dv fixed to $1.5{\cdot}10^{-8}$ m²/s |
| | **AstroSticks** | Pseudo-diffusivity: dv | $9{\cdot}10^{-9}$ m²/s ≤ dv ≤ $1{\cdot}10^{-7}$ m²/s<br>or dv fixed to $4{\cdot}10^{-8}$ m²/s |
| **Intracellular** (fraction **fic**) | **Sphere** | Radius: $R_{Sph}$ | $1{\cdot}10^{-7}$ m ≤ $R_{Sph}$ ≤ $2{\cdot}10^{-5}$ m |
| | | Intrinsic diffusivity: Dic | $1{\cdot}10^{-11}$ m²/s ≤ Dic ≤ $3{\cdot}10^{-9}$ m²/s |
| **Free Water** (fraction **ffw**) | **Ball** | Diffusivity: $D_{FW}$ | $D_{FW}$ fixed to $3{\cdot}10^{-9}$ m²/s |

**Supplementary Table S2**: Details of patients' characteristics.

| Patient no. | Age | Sex | Biopsy/surgery | Histology diagnosis | WHO grade | IDH mutation |
|---|---|---|---|---|---|---|
| 01 | 69 | F | Biopsy | Astrocytoma | 3 | wild-type |
| 02 | 61 | M | Surgery | Astrocytoma | 3 | wild-type |
| 03 | 65 | M | Biopsy | Glioblastoma | 4 | wild-type |
| 04 | 71 | M | Surgery | Melanoma metastasis | / | - |
| 05 | 41 | M | Biopsy | Astrocytoma | 3 | mutated |
| 06 | 24 | M | Surgery | Astrocytoma | 2 | wild-type |
| 07 | 57 | M | Surgery | RNa (Pulmonary AdenoCa Met) | / | / |
| 08 | 74 | M | Surgery | Glioblastoma | 4 | wild-type |
| 09 | 73 | M | Surgery | Astrocytoma | 2 | wild-type |
| 10 | 20 | M | Surgery | Ependymoma | 3 | / |
| 11 | 31 | F | Biopsy | Astrocytoma | 2 (3)b | wild-type |
| 12 | 58 | M | Biopsy | Glioblastoma | 4 | wild-type |
| 13 | 77 | M | Surgery | Glioblastoma | 4 | wild-type |
| 14 | 65 | M | Surgery | Melanoma metastasis | / | / |
| 15 | 32 | F | Surgery | Subependymoma | 1 | / |
| 16 | 51 | M | Surgery | Astrocytoma | 3 | mutated |
| 17 | 65 | M | Surgery | Glioblastoma | 4 | wild-type |
| 18 | 64 | F | Surgery | Astrocytoma | 3 | mutated |
| 19 | 19 | M | Surgery | Astrocytoma | 2 | mutated |
| 20 | 42 | M | Surgery | Focal cortical dysplasia | - | - |
| 21 | 37 | F | Surgery | Astrocytoma | 2 | wild-type |

[a] RN = radionecrosis
[b] biopsy-confirmed WHO 2 with foci of radiological progression

**Supplementary Table S3**: Ranking of two- and three-compartment models fitted on high-b signals, based on the average AICc across patients. A single-Ball model is also included for comparison.

| **Model ranking in the tumour core** | **Model ranking in the peritumoural ROI** |
|---|---|
| Zeppelin-Sphere | Zeppelin-Tensor-Sphere |
| Zeppelin-Cylinder-Sphere | Tensor-Sphere |
| Zeppelin-Tensor-Sphere | Ball-Tensor-Sphere |
| Zeppelin-Stick-Sphere | Zeppelin-Cylinder-Sphere |
| Zeppelin-WatsonSticks-Sphere | Zeppelin-Stick-Sphere |
| Tensor-Sphere | Zeppelin-Sphere |
| Ball-Tensor-Sphere | Zeppelin-WatsonSticks-Sphere |
| Ball-Stick-Sphere | Ball-WatsonSticks-Sphere |
| Ball-Zeppelin-Sphere | Ball-Zeppelin-Sphere |
| Ball-WatsonSticks-Sphere | Ball-Stick-Sphere |
| Ball-Cylinder-Sphere | Ball-Cylinder-Sphere |
| Ball-Sphere | Ball-Sphere |
| Cylinder-Sphere | Cylinder-Sphere |
| Ball | Ball |
| Stick-Sphere | Stick-Sphere |

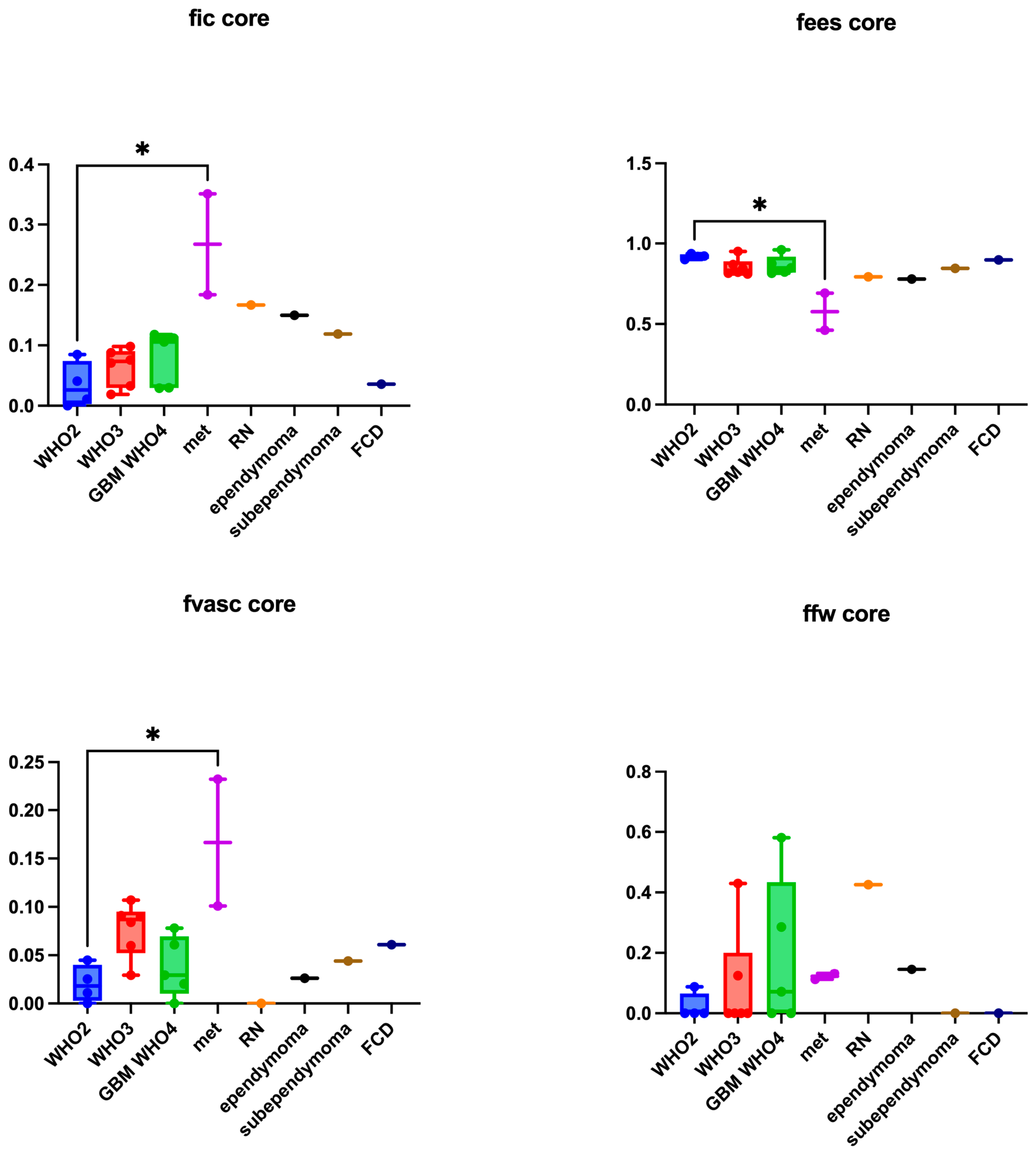


**Supplementary Figure S1.** VERDICT results in the tumour core for the whole cohort of patients. The boxplots show the ROI median values in each patients group for the signal fraction of each compartment. Statistically significant differences are marked (* $p < 0.05$).

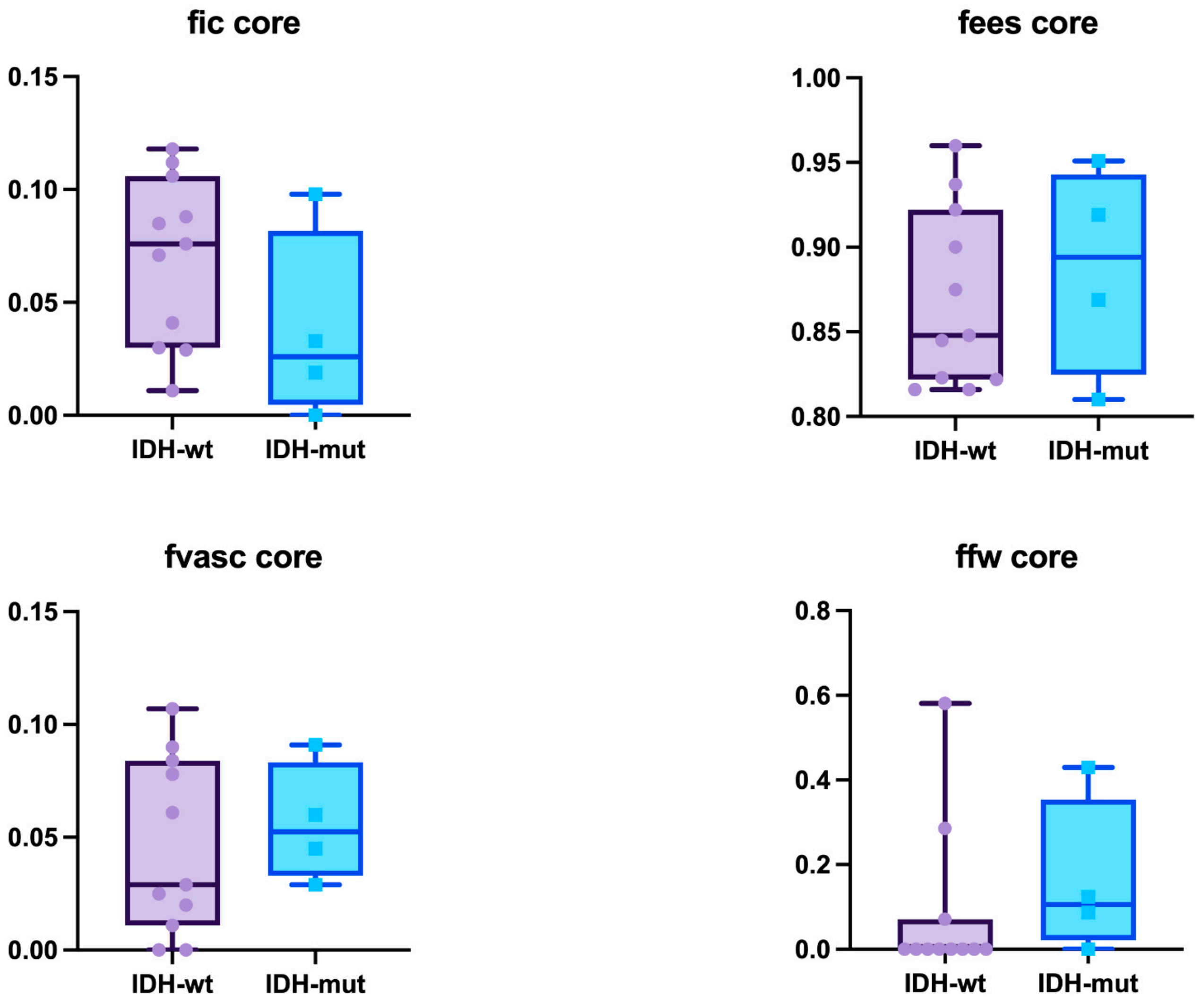


**Supplementary Figure S2.** VERDICT results in the tumour core according to IDH status. The boxplots show the ROI median values in each patients group for the signal fraction of each compartment.
